# Single NIR Laser-Activated Multifunctional Nanoparticles for Cascaded Photothermal and Oxygen-Independent Photodynamic Therapy

**DOI:** 10.1007/s40820-019-0298-5

**Published:** 2019-08-19

**Authors:** Xiaomin Li, Yang Liu, Fei Fu, Mingbo Cheng, Yutong Liu, Licheng Yu, Wei Wang, Yeda Wan, Zhi Yuan

**Affiliations:** 1 Key Laboratory of Functional Polymer Materials of Ministry of Education, Institute of Polymer Chemistry, Collaborative Innovation Center of Chemical Science and Engineering (Tianjin), Nankai University, Tianjin, 300071 People’s Republic of China; 2 Department of Radiology, Tianjin Hospital, Tianjin, 300210 People’s Republic of China

**Keywords:** Bi_2_Se_3_, Free radical, Oxygen-independent, Photothermal, Photodynamic

## Abstract

Inconvenient dual-laser irradiation and tumor hypoxic environment as well as limited judgment of treating region have impeded the development of combined photothermal and photodynamic therapies (PTT and PDT). Herein, Bi_2_Se_3_@AIPH nanoparticles (NPs) are facilely developed to overcome these problems. Through a one-step method, free radical generator (AIPH) and phase transition material (lauric acid, LA, 44–46 °C) are encapsulated in hollow bismuth selenide nanoparticles (Bi_2_Se_3_ NPs). Under a single 808-nm laser irradiation at the tumor area, hyperthermia produced by Bi_2_Se_3_ not only directly leads to cell death, but also promotes AIPH release by melting LA and triggers free radical generation, which could further eradicate tumor cells in hypoxic environments. Moreover, Bi_2_Se_3_ with high X-ray attenuation coefficient endows the NPs with high computed tomography (CT) imaging capability, which is important for treating area determination. The results exhibit that Bi_2_Se_3_@AIPH NPs possesses 31.2% photothermal conversion efficiency for enhanced PTT, ideal free radical generation for oxygen-independent PDT, and 37.77 HU mL mg^−1^ X-ray attenuation coefficient for CT imaging with high quality. Most importantly, the tumor growth inhibition rate by synergistic PTT, PDT, and following immunotherapy is 99.6%, and even one tumor disappears completely, which demonstrates excellent cascaded synergistic effect of Bi_2_Se_3_@AIPH NPs for the tumor therapy.

**Electronic supplementary material:**

The online version of this article (10.1007/s40820-019-0298-5) contains supplementary material, which is available to authorized users.

## Highlights

- Bi_2_Se_3_@AIPH nanoparticles (NPs) can realize photothermal and photodynamic therapies in a cascading manner when exposed to “one” single NIR laser.
- Hyperthermia produced by Bi_2_Se_3_ NPs under NIR laser irradiation cannot only be used for photothermal therapy, but also the “switch” for controlled release of AIPH and excitation of free radical at the tumor site.
- Free radical produced by thermal-responsive decomposition of AIPH is oxygen-independent, which treats hypoxic tumor well and further enhances immune response.

**Electronic supplementary material:**

The online version of this article (10.1007/s40820-019-0298-5) contains supplementary material, which is available to authorized users.

## Graphical abstract

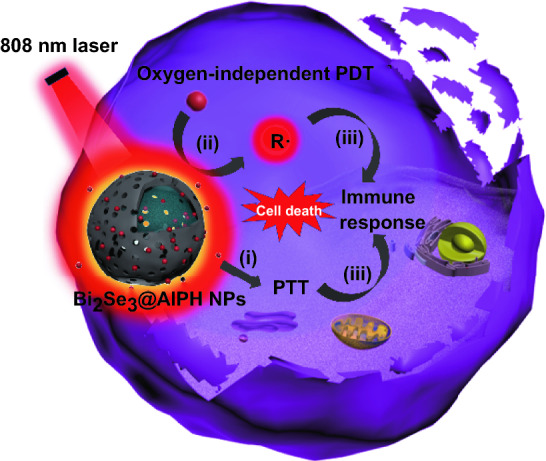

## Introduction

Phototherapy, mainly photothermal therapy (PTT) and photodynamic therapy (PDT), holds a huge promise in biomedical field due to high spatiotemporal precision and non-invasive properties [1–9]. Although both make use of light energy, PTT and PDT are different. PTT agents absorb light and convert it into heat to kill cancer cells [4], while photosensitizer in PDT absorbs light to produce toxic reactive oxygen species for cancer cells in the presence of oxygen [10]. Many strategies have been proposed to improve PTT efficiency (enhancing the cumulative dosage of tumor area [11, 12], applying appropriate laser dosage [13, 14], guiding treatment via imaging [15–18], increasing photothermal conversion efficiency [19, 20], etc.) or PDT outcomes (choosing proper photosensitizer [21, 22], indirect excitation [10, 23, 24], supplying oxygen externally [25–28], etc. [29, 30]).

Recently, it has been demonstrated that one phototherapy combined with others has showed enhanced anticancer efficacy compared with sole PTT or PDT, especially the synergistic PTT and PDT [31–33]. Xueji Zhang et al. designed a sort of porphyrin derivatives conjugated with graphene quantum dots, which can yield ^1^O_2_ by 635 nm laser and have a good photothermal effect with 25.58% photothermal conversion efficiency by 980 nm laser [34]. Shaojun Guo et al. constructed a black phosphorus (BP)-based drug delivery system which can achieve pH-/photo-responsive drug release, ^1^O_2_ generation by 660 nm laser, and photothermal activity by 808 nm laser [35]. Hongjie Zhang et al. reported biocompatible copper ferrite nanospheres with enhanced ROS production under irradiation with a 650 nm laser through direct electron transfer and photoenhanced Fenton reaction, and high photothermal conversion efficiency upon exposure to an 808 nm laser [36]. Although synergistic antitumor efficacy is improving, the employment of two lasers is more inconvenient to operate and more expensive to bear. Thus, some studies have tried the combined phototherapy using one laser [13, 37, 38]. Pingping Yang et al. introduced a therapeutic nanoplatform (named IONCs@Ce6-DOX/PCM) which loaded the photosensitizer chlorin e6 in amine-functionalized iron oxide nanocrystals (IONCs), and synergistic treatment efficiency has been verified when irradiated by single 650-nm laser [39]. Xinghua Xia et al. carefully chose photosensitizers indocyanine green modified on the Au/MoS_2_ hybrid via hydrophobic interactions and *π* − *π* stacking; under single 808-nm laser activation, the proposed strategy of simultaneous PDT/synergistic PPTT effectively reduces the treatment time and achieves high therapeutic index [13]. This ingenious cooperation of photosensitizers and PTT agents has exhibited great antitumor capabilities under the exposure of a single laser, but another problem emerged. Despite the selection of a suitable photosensitizer, photosensitizer-induced PDT severely depends on oxygen which is seriously deficient in the tumor area, which restricts the final efficiencies of most phototherapies [4, 30]. Thus, finding other ways to overcome these problems is significant.

Free radical initiator, usually used in free radical polymerization, has been verified to produce free radical through thermal decomposition [40, 41]. Youhan Xia et al. first reported this oxygen-independent free radical for eradicating cancer cells as a PDT agent [42], and Xianzheng Zhang et al. further demonstrated the killing mechanism to tumor cells under different conditions and its application in hypoxic tumor [43, 44]. Thus, employing this kind of free radical initiator in PDT to surpass tumor has showed bright future in the cancer therapy [45]. And in the process of initiator decomposition, production rate of free radical is thermal-dependent [40, 41]. This thermal energy can be provided by PTT agents through light irradiation, and one laser can meet the demand of combined therapy, which perfectly avoids the laser problem. And to guarantee rapid generation of sufficient free radical in PDT and photothermal effect in PTT to treat the tumor, hyperthermia is needed. Furthermore, guiding the treatment via imaging techniques especially CT imaging is crucial for phototherapy. Frustratingly, it is often dragged down by restricted X-ray attenuation coefficient of diagnostic agents [4]. In a word, a proper PDT & PTT carrier is expected to meet the following demands: (1) high loading capacity to carry enough initiators; (2) high photothermal conversion to produce hyperthermia and activate free radical; and (3) high imaging capability to precisely judge the tumor region.

**Scheme 1:**
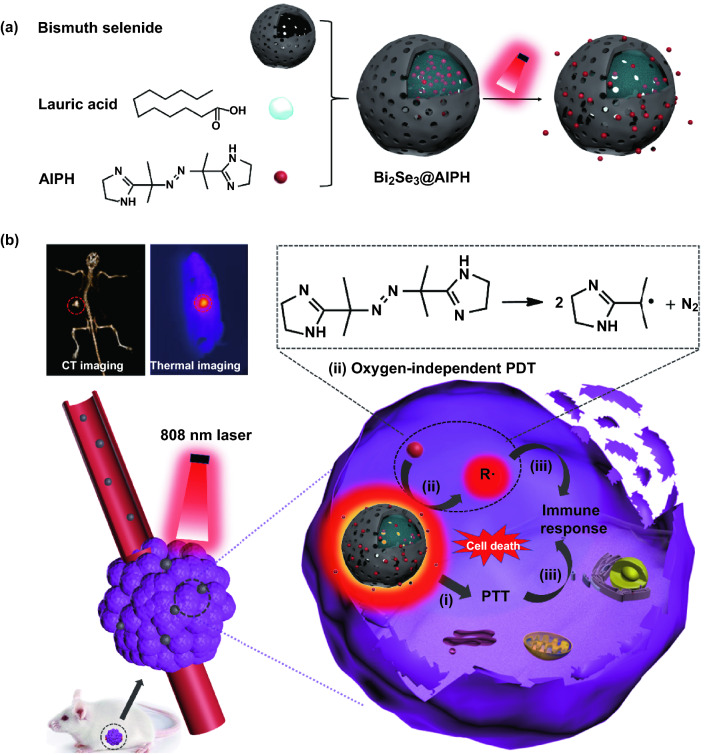
Schematic illustration of Bi_2_Se_3_@AIPH as single 808-nm laser-activated nanoparticles for tumor therapy: **a** synthetic route and release process of Bi_2_Se_3_@AIPH; **b** theranostic process for CT and thermal imaging-guided cascaded photothermal, oxygen-independent photodynamic therapies along with immune response

Among all kinds of PTT agents, hollow bismuth selenide nanoparticles (Bi_2_Se_3_ NPs) with high X-ray attenuation coefficient, high loading space, and high photothermal conversion capability reported by Rong Chen et al. and Miao Yu et al. perfectly meet all the requirements above and it also possesses other advantages, such as cheap, synthetically convenient, and biocompatible [46, 47]. Herein, we employed hollow Bi_2_Se_3_ NPs and internally loaded with 2,2-azobis[2-(2-imidazolin-2-yl) propane] dihydrochloride (AIPH, free radical initiator) and lauric acid (LA, phase change material, 44–46 °C), which is labeled as Bi_2_Se_3_@AIPH to realize the synergistic therapies (Scheme 1). When circulating in the blood, AIPH remains inside to avoid side effect caused by AIPH leakage due to the use of phase transition material. When exposure to a single 808-nm laser, Bi_2_Se_3_@AIPH NPs can not only kill tumor cells directly, but also release AIPH and accelerate the production of toxic free radical in a cascading manner. Moreover, Bi element in the Bi_2_Se_3_@AIPH theoretically possesses improved CT imaging capability than the commonly used CT contrast agents, which is better for determining the tumor area before the treatment. In vitro *and* in vivo CT imaging experiments were performed to assess CT imaging capability of Bi_2_Se_3_@AIPH NPs. Then, the hyperthermia was characterized by the temperature change via thermal imaging system, and the free radical decomposed from AIPH was detected by in vivo fluorescence imaging system. Finally, in vitro cytotoxicity and in vivo anticancer efficacy were examined to demonstrate the superiority of this CT imaging-guided cascaded synergistic effect.

## Experimental Section

### Materials and Measurements

#### Materials

Bi(NO_3_)_3_·5H_2_O, HNO_3_, NaOH, polyvinylpyrrolidone (PVP), ethylene glycol (EG), Na_2_SeO_3_, ascorbic acid, 2,2-azobis[2-(2-imidazolin-2-yl)propane] dihydrochloride (AIPH), lauric acid (LA) were purchased from Tianjin Heowns Co., Ltd. 2, 2′-azino-bis(3-ethylbenzothiazoline-6-sulfonic acid) (ABTS), 5,5-Dimethyl-1-pyrroline N-oxide (DMPO), α-(4-Pyridyl 1-oxide)-N-tert-butylnitrone (POBN), 5,5′-Dithiobis (2-nitrobenzoic acid) (DTNB) were purchased from Tianjin Sulai Co., Ltd. Lysotracker Green, 4′,6-diamidino-2-phenylindole (DAPI), 2′, 7′-dichlorofluorescein diacetate (DCFH-DA), 3-(4,5-dimethyl-2-thiazolyl)-2,5-diphenyl-2-H-tetrazolium bromide (MTT), acridine orange/ethidium bromide(AO/EB) staining kit, dihydrorhodamine were purchased from Tianjin Solomon Biotech, Inc. TNF-α mouse ELISA Kit and IFN-γ mouse ELISA Kit were purchased from Thermo Fisher Scientific (Waltham, MA, USA). The water used in this work was provided by Millipore Elix System (Millipore, Bedford, MA).

Human hepatoma HepG2 cells were cultured in Dulbecco’s minimal essential medium (DMEM) containing 10% FBS in a standard cell culture environment (humidified, 37 °C, 5% CO_2_). The initial cell densities at 96-well and 24-cell plates are 8000 and 25,000 cells/well, respectively.

Imprinting control region mice (ICR, male, 6–8 weeks) were provided by Beijing Vital River Laboratory Animal Technology Co., Ltd. All the experiment procedures related to the ICR mice were carried out in strict compliance with the “Guide for the Care and Use of Laboratory Animals.” The tumor xenograft model is established by subcutaneously injecting mice H22 liver tumor cells (5 × 10^7^ cells mL^−1^, 0.1 mL) into the right oxter of ICR mice.

#### Measurements

The morphology and size were measured by transmission electron microscopy (TEM, FEI, USA). The hydrodynamic diameters were detected by dynamic light scattering (DLS, Zetasizer Nano ZS90, UK); absorption spectra were recorded by UV-2550 (Shimadzu, Japan). X-ray diffraction (XRD, D/max-2500, Japan) measurement was taken to determine the crystalline phases present in the Bi_2_Se_3_ powders. Thermogravimetric analysis (TGA) is conducted by thermal analysis system (NETZSCH, Germany). The light source for detection and treatment was provided by 808 nm laser (WG1533D6, Ainajie, China), and the thermal imaging is achieved by a thermal imaging system (Fotric 220, ZXF, USA).

### Synthesis of Bi_2_Se_3_@ AIPH NPs

AIPH (0.2 g) and LA (0.15 g) were dissolved in mixed solvent (DI water/methanol = 1:1). And then, 10 mg Bi_2_Se_3_ powders were added into the mixture and continued to react for 3 days. The obtained Bi_2_Se_3_@AIPH solution was centrifuged for several times. And the final product was dried by lyophilization and placed in a dryer for further use.

### Photothermal Evaluation

To investigate the photothermal effect of Bi_2_Se_3_ and Bi_2_Se_3_@AIPH NPs, we measured the thermal images and temperature changes in the materials at different concentrations (0, 0.01, 0.05, 0.1, and 0.2 mg mL^−1^) by the irradiation of 808 nm laser (1 W cm^−2^, 5 min). And the NPs were both measured three times, respectively. The photograph and the temperature were recorded every half minute. After that, we also measured the cycle ability using 0.2 mg mL^−1^ Bi_2_Se_3_@AIPH as the representative. In brief, the material was irradiated for 5 min under the same conditions (“on” state) at first, then naturally cooled down to room temperature (“off” state), which is called one cycle. Five “on and off” cycles were measured, and the temperatures were also recorded every half minute.

### AIPH Loading Capacity

To measure the total loading amount of AIPH and LA, the TGA of Bi_2_Se_3_@AIPH was carried out. And the loading amount of AIPH was calculated by an indirect method. Briefly, AIPH solutions at different concentrations (1, 2, 5, 8, and 10 mg mL^−1^) were prepared and measured by UV–Vis spectrum. The standard curve of it was calculated as *y* = 0.06516*x* + 0.09777 (*R*^2^ = 0.99999). And the AIPH in the supernatant after the centrifugation was measured by UV–Vis spectrum, and the amount in supernatant was determined through the standard curve of AIPH. The loading amount of AIPH was obtained by subtraction method of weight.

### AIPH Release

The thermo-responsive release behavior is evaluated by intermittent exposure to 808 nm laser. The Bi_2_Se_3_@AIPH NPs are irradiated every half hour, and every irradiation lasts for 5 min per exposure to 808 nm laser. The control group is the releasing AIPH percentage of the Bi_2_Se_3_@AIPH NPs at 37 °C in dark environment without irradiation. The separated AIPH solution is obtained by centrifugation (8000 *r*, 20 min). The releasing amounts of AIPH at different time points were detected by UV–Vis spectrum. And the content of AIPH at each point was calculated according to the standard curve of AIPH, and the weight percentage was determined by these results.

### Free Radical Detection

The releasing AIPH is thermolabile, and one AIPH would be decomposed into two alkyl free radicals, which was measured by two methods. One is based on the reaction of ABTS and radical, and the experiment process was conducted according to the literature [42]. The absorbance of the obtained ABTS^+·^ under 5 min irradiation (1 W cm^−2^) was detected by UV–Vis spectrum every one minute. The other one is based on the capture of free radical by radical spin traps (DMPO and POBN) [43], and the paramagnetic signals (mainly electron spin resonance signals) of captured products can be detected by electron paramagnetic spectrometer (MS400, Magnettech, Germany). Briefly, 0.03 mg mL^−1^ Bi_2_Se_3_@AIPH solutions were mixed with 100 mM DMPO and POBN solution under air and N_2_ atmosphere, respectively. After the irradiation of 808 nm laser (1 W cm^−2^), the paramagnetic signal was detected by an electron paramagnetic spectrometer.

### In Vitro Photothermal Effect

The thermal effect of Bi_2_Se_3_@AIPH on the HepG2 cells is measured by thermal imaging system. Similarly, the HepG2 cells were incubated for 4 h in 96-well plates. After washing with PBS three times, the 808 nm laser was used to irradiate the related wells (1 W cm^−2^, 5 min), and the thermal pictures were recorded by thermal imaging system.

### Intracellular Free Radical Assessment

To investigate the generation of free radical in the HepG2 cells, the DCFH-DA assay was employed. Zinc phthalocyanine (ZnPc) which can only produce ^1^O_2_ under the normoxic environment was employed as a comparison. ZnPc and LA, same amount as AIPH and LA, similarly loaded in the hollow Bi_2_Se_3_ NPs according to the “Synthesis of Bi_2_Se_3_@ AIPH NPs.” Then, the HepG2 cells were cultured in the 24-well plate for 24 h, the Bi_2_Se_3_@AIPH (40 μg mL^−1^) and Bi_2_Se_3_@ZnPc (40 μg mL^−1^) divided into normoxia and hypoxia groups were added in the plate and incubated for another 4 h, followed by PBS washing for three times. Then, the HepG2 cells were irradiated by the laser (Bi_2_Se_3_@AIPH group: 808 nm laser, 0.7 W cm^−2^, 5 min; Bi_2_Se_3_@ZnPc group: 808 nm laser, 0.7 W cm^−2^, 5 min and then 660 nm laser, 300 mW cm^−2^, 3 min), followed by incubation with 10 μM DCFH-DA for 30 min. The wells were imaged by CLSM and quantified by microplate reader. The hypoxic condition was realized by using the culture medium with 100 μM CoCl_2_ solution [42, 45].

### Intracellular GSH Evaluation

The HepG2 cells were seeded in the 24-well plate and incubated for 24 h. Bi_2_Se_3_@AIPH NPs (40 μg mL^−1^, normoxia and hypoxia group) were added into the plates, respectively. After another 4 h incubation, the cells were irradiated by 808 nm laser (1 W cm^−2^, 5 min). Then, the cells were washed by PBS three times and 200-μL Triton-X-100 lysis buffer (0.4%) was added at each well. The lysates were centrifuged, and the supernatant (50 μL) was mixed with 200 μL of Ellman’s reagent (0.5 mM DTNB) for 30 min. And the absorption was measured by microplate reader.

### Cytotoxicity and Apoptosis

To access the cell membrane destruction and cytotoxicity of Bi_2_Se_3_@AIPH NPs with/without NIR irradiation in normoxic or hypoxic condition, the MDA, MTT, and AO/EB assays were used. The HepG2 cells were plated in 96-well plate and cultured for 24 h. PBS, AIPH, Bi_2_Se_3_, and Bi_2_Se_3_@AIPH (40 μg mL^−1^) were added into the wells and incubated for another 4 h. After being washed by PBS for three times, the HepG2 cells were irradiated by 808 nm laser for 5 min and incubated for 12 h. And the following process is strictly in accordance with standard operation manual of MDA measurement, MTT assay, and AO/EB staining. The absorbance in every well was measured by microplate reader, and fluorescence imaging of the cells was recorded by CLSM.

### Biodistribution of Bi Element

When the tumor size on the ICR mice reached 120 mm^3^, the mice (*n* = 3) were intravenously injected with Bi_2_Se_3_@AIPH (2 mg mL^−1^, 100 μL). The mice were sacrificed at 3, 6, and 24 h after the injection. The main organs (heart, liver, spleen, lung, and kidney) and tumors were weighed and dissolved in aqua regia, respectively. After sufficient dissolution and centrifugation, the solutions were diluted to measure the Bi element concentration by inductively coupled plasma-atomic emission spectrometry (ICP-AES).

### CT Imaging

To study the in vitro CT imaging, a series of the Bi_2_Se_3_@AIPH NPs and iohexol with different concentrations (1, 10, 20, 30, 40, and 50 mg mL^−1^) was prepared. The CT effect was imaged by a CT imaging system (GE Discovery 750 HD, Tianjin Hospital). The test is under the condition of 200 mA and 120 kV.

To conduct in vivo CT imaging, ICR mice bearing H22 tumors were employed. When the tumor size on the ICR mice reached 120 mm^3^, the mice were divided into two groups and intratumorally injected with Bi_2_Se_3_@AIPH NPs and iohexol (10 mg mL^−1^, 75 μL), respectively. After 20 min, the mice were imaged by a GE Discovery 750 HD medical system. Every group has three mice. What’s more, the mice were intravenously injected with Bi_2_Se_3_@AIPH NPs (2 mg mL^−1^, 100 μL); the CT imaging before and after (3, 6, and 24 h) intravenous injection was also studied by the above CT imaging system under the same condition. The data post-process was in the same parameter condition including window width and window position.

### In Vivo Thermal Imaging

When the tumor size on the ICR mice reached 120 mm^3^, the mice were divided into four groups (*n* = 4). 100 μL PBS, AIPH (2 mg mL^−1^), Bi_2_Se_3_ (2 mg mL^−1^), and Bi_2_Se_3_@AIPH (2 mg mL^−1^) were intravenously injected into the mice. After 24 h, the mice were irradiated by 808 nm laser (1 W cm^−2^, 5 min) and imaged by thermal imaging system every 1 min.

### In Vivo Free Radical Detection

The free radical was detected via the reduction in dihydrorhodamine. When the tumor size on the ICR mice reached 120 mm^3^, the mice were divided into two groups. The mice were injected with Bi_2_Se_3_@AIPH (2 mg mL^−1^, 100 μL) via tail vein. After 24 h, the hair was shaved, then one group is irradiated by 808 nm laser (1 W cm^−2^, 5 min), and the other is not irradiated. Then, both of them were intratumorally injected with 20 mM dihydrorhodamine. The fluorescent signal generated from rhodamine in the mice was detected by in vivo fluorescence imaging system (IVIS Lumina II, Caliper Life Sciences, MA, USA). Then, the mice were sacrificed and the tumor was collected for ex vivo fluorescence imaging. The quantitative fluorescent values in vivo and ex vivo tumor were also calculated.

### In Vivo Antitumor Study

The mice were randomly divided into four groups (PBS, AIPH, Bi_2_Se_3_, and Bi_2_Se_3_@AIPH). Each group has four mice. After the tumor size reached 120 mm^3^, 100 μL PBS, AIPH (2 mg mL^−1^), Bi_2_Se_3_ (2 mg mL^−1^), and Bi_2_Se_3_@AIPH (2 mg mL^−1^) were injected into the mice via tail vein. Before the treatment, the hair was shaved for better laser penetration depth. Then, the mice were irradiated by 808 nm laser (1 W cm^−2^, 5 min) after 24 h accumulation in tumor. The body weight and the tumor size were measured every 2 days. At 14 days, all the mice were sacrificed and the tumors were pictured.

### Histology Analysis

At the end of the treatment (day 14), the mice were sacrificed by cervical dislocation, and the major organs (heart, liver, spleen, lung, and spleen) were taken out and washed with PBS several times, and finally fixed in 4% paraformaldehyde. According to standard protocols of hematoxylin & eosin (H&E) staining, the sections were pictured by optical microscope (IX53, Olympus, Japan).

### Cytokine Test

The blood was taken from the eyes of the mice after the inoculation of tumor cells in mice and during the treatment every 3 days. Each group has three mice. Then, the serums were obtained by the centrifugation (1000 *r*, 10 min, 4 °C). The tumor necrosis factor-α (TNF-α) and interferon-γ (IFN-γ) in the separated serums were measured following the procedure described in the TGF-α mouse ELISA Kit and IFN-γ mouse ELISA Kit.

### Statistical Analysis

The results are representative of replicate experiments and are presented as the mean value with standard deviation (mean ± SD). Student’s *t* test was used to compare the differences in the results. *p < 0.05 was considered statistically significant. **p < 0.01 was considered extremely significant.

## Results and Discussion

### Characterization of Bi_2_Se_3_@/AIPH NPs

**Fig. 1:**
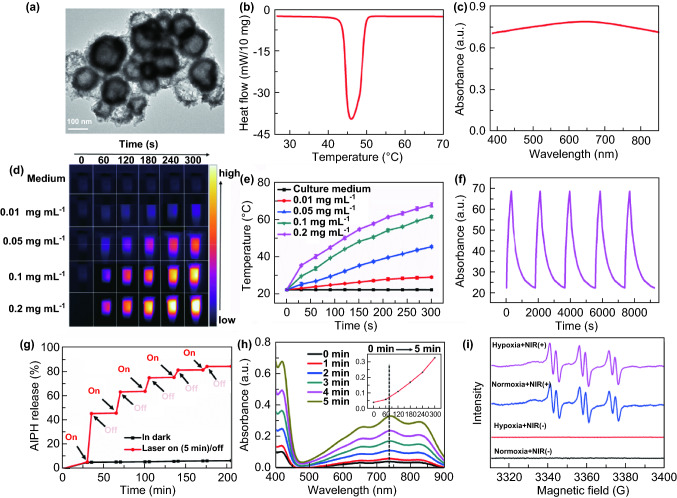
Characterization of Bi_2_Se_3_@AIPH NPs and measurements of photothermal and free radical properties. **a** TEM images, **b** DSC curve, and **c** UV–Vis spectrum of Bi_2_Se_3_@AIPH. **d** Representative photothermal images of Bi_2_Se_3_@AIPH solution at the concentrations of 0, 0.01, 0.05, 0.1, and 0.2 mg mL^−1^ (1 W cm^−2^, 5 min). **e** Corresponding temperature change evaluation at various concentrations. *n* = 3 **f** Cycle stability of Bi_2_Se_3_@AIPH (0.2 mg mL^−1^ Bi_2_Se_3_@AIPH solution as the representative). **g**. Thermo-responsive release profiles of AIPH from Bi_2_Se_3_@AIPH NPs, and the ‘‘on’’ switch represents the NPs are irradiated for 5 min (1 W cm^−2^) by 808 nm laser, and the ‘‘off’’ means the irradiation stops. Except the irradiation, the NPs solution was placed in the environment of 37 °C. **h** Absorbance of ABTS^+·^ generated from the reaction of ABTS and Bi_2_Se_3_@AIPH under 0–5 min irradiation (1 W cm^−2^, 5 min). **i** electron spin resonance (ESR) spectrum of 50 mM POBN in 0.1 mg mL^−1^ Bi_2_Se_3_@AIPH with or without irradiation at normoxic and hypoxic atmosphere

Synthesis of Bi_2_Se_3_ NPs is achieved via hydrothermal method using Bi_2_O_3_ as the bismuth precursor and template. The TEM images and hydrodynamic diameters of Bi_2_O_3_ and Bi_2_Se_3_ are displayed in Figs. S1 and S2, and the result shows Bi_2_O_3_ NPs is spherical with the hydrodynamic diameters of 91.28 nm, and by the reduction in ascorbic acid and sodium selenite (a selenite source), this template forms hollow Bi_2_Se_3_ and the average hydrodynamic diameters are about 122.4 nm, which is slightly larger than that of Bi_2_O_3_. And the crystallization nature of Bi_2_Se_3_ was characterized by XRD as shown in Fig. S3. All the diffraction peaks in Fig. S3 can be well indexed to Bi_2_Se_3_ (JCPDS No. 12-732), which demonstrates the high purity of as-prepared Bi_2_Se_3_ NPs. The photothermal ability of Bi_2_Se_3_ NPs was also studied, and the result (Fig. S4) shows that Bi_2_Se_3_ NPs has good photothermal performance. (The temperature of 0.2 mg mL^−1^ Bi_2_Se_3_ NPs solution can rise up to 69.2 °C in 5 min.) Then, AIPH and LA were simultaneously encapsulated in the Bi_2_Se_3_ NPs in a simple one-step method. The TEM image in Fig. 1a clearly reveals the hollow morphology of Bi_2_Se_3_@AIPH. And the hydrodynamic diameter in Fig. S5 measured by DLS is 141.2 nm, which is bigger than that of Bi_2_Se_3_. And the hydrodynamic diameters basically keep the same after 1 week and 1 month, which also verifies the stability of Bi_2_Se_3_@AIPH NPs. Moreover, by TGA in Fig. S6, the total loading amount of AIPH and LA is 17.7 wt%. And by measuring the amount of the unloaded AIPH and the subtraction method in Fig. S7, the loading amounts of AIPH are 10.2 wt%. Given its high AIPH loading, more free radicals are expected to produce to kill tumor cells. And the DSC curve in Fig. 1b not only confirms the successful loading of LA, but also show the melting point of LA in Bi_2_Se_3_@AIPH is 45.87 °C (peak value), which is a little lower than the pure LA. (Peak value is 46.89 °C; data are not shown.) When the temperature is above the melting point, LA could dissolve and flow, accelerating the release of others in the NPs. While lower than 45.87 °C, the AIPH could keep inside and not release. Besides, the UV–Vis spectrum of the Bi_2_Se_3_@AIPH in Fig. 1c exhibits a broad absorption in ultraviolet–visible–near-infrared area. The strong absorption in near-infrared (NIR) window endows Bi_2_Se_3_@AIPH NPs with ability of photothermal conversion, which is crucial in PTT.

### Light-Activated Photothermal and Free Radical Effects

After the irradiation of 808 nm laser, the double effects (photothermal and thermal-triggered free radical generation) were investigated. First, to investigate the photothermal ability of the material, Bi_2_Se_3_@AIPH NPs with different concentrations (0, 0.01, 0.05, 0.1, and 0.2 mg mL^−1^) were irradiated by 808 nm laser (1 W cm^−2^) for 5 min, and the thermal images and temperature changes were recorded by the thermal imaging system. As displayed in Fig. 1d, e, it is verified that the temperature change in Bi_2_Se_3_@AIPH solution was concentration- and irradiation duration-dependent. At the same irradiation time, using the data at 5 min as the example, the solution temperature is increasing along with the increase in concentration of Bi_2_Se_3_@AIPH NPs, and 0.2 mg mL^−1^ Bi_2_Se_3_@AIPH solution could even reach up to 68.6 °C. Moreover, the photothermal conversion efficiency (*η*) of Bi_2_Se_3_@AIPH is calculated to be ~ 31.2%, which is higher than that of the commonly used PTT agents, such as gold rod (21%) [48] and gold nanoshell (17.5%) [49]. And the cycling photostability of Bi_2_Se_3_@AIPH NPs is also tested by irradiating 0.2 mg mL^−1^ Bi_2_Se_3_@AIPH solution for five times, and the results in Fig. 1f show no temperature decrease at every irradiation, which guarantees its good photothermal stability in PTT.

Importantly, the irradiation of the NIR laser not only endows the material with the excellent thermal effect but also the power to release AIPH and generate free radical. The AIPH releasing experiment had carried out, and the result in Fig. 1g shows that when Bi_2_Se_3_@AIPH NPs are irradiated by the 808 nm laser, the AIPH release is increasing, while when the laser is off, the increase in release amount is hardly seen in the picture. And only a small amount of AIPH (205 min release percentage: 6.8%) was released from the Bi_2_Se_3_@AIPH NPs at the physiological environment (37 °C) without irradiation. These results verify that the LA could block the AIPH release well without irradiation, which could avoid the AIPH leakage in the blood circulation. Under the irradiation, the releasing amount of the AIPH is ever-increasing along with the increase in irradiation duration and is capable of reaching 80% after several irradiations. Next, the generated free radicals were detected by UV–Vis and ESR spectrum. 2, 2′-azino-bis(3-ethylbenzothiazoline-6-sulfonic acid) (ABTS) can react with free radicals and form relatively stable ABTS^+·^ which can be detected by UV–Vis spectrum [42, 45, 50]. As shown in Fig. 1h, the characteristic absorbance at 500–900 nm reflects the production of ABTS^+·^, and along with prolonged exposure to NIR laser (0–5 min), the peak intensity at 736 nm is obviously increasing (seeing the insertion), which demonstrates that the loading AIPH could generate large amount of free radicals at 5 min irradiation. On the other hand, to guarantee its feasibility in hypoxic tumor, we also hired two free radical spin probes (POBN and DMPO) to trap the generated free radical under normoxic and hypoxic atmosphere. As shown in Figs. 1i and S8, whether with or without O_2_, it is clear that characteristic signals have appeared under laser irradiation, while no signal is found without irradiation in both conditions. For POBN, the characteristic peaks were similar under normoxic and hypoxic condition. While for DMPO, the peak is different, but it also verifies the existence of free radical, similar to the literature [43]. In a word, whether it is under normoxic or hypoxic atmosphere, free radical can be generated under the irradiation.

### Performance of Bi_2_Se_3_@AIPH NPs in HepG2 Cells

**Fig. 2:**
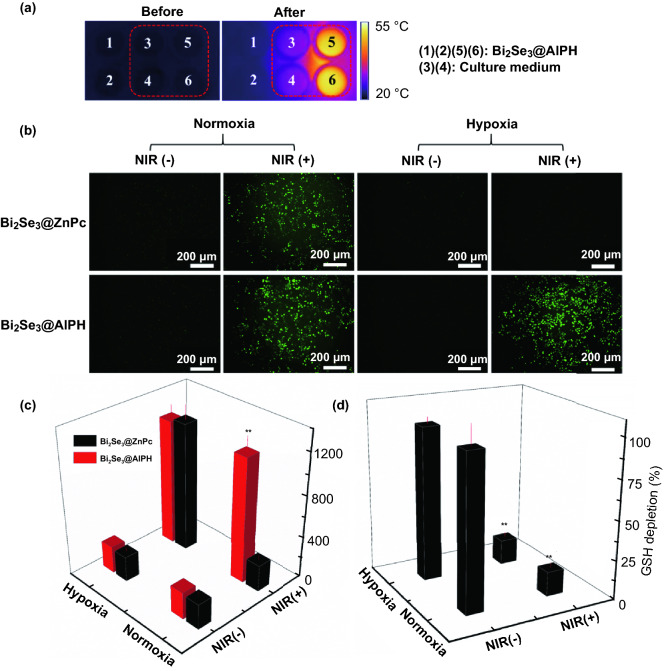
In vitro photothermal and free radial effect. **a** Thermal images of HepG2 cell culture medium in 96-well plate: (1), (2), (5), and (6) were added with Bi_2_Se_3_@AIPH NPs, while (3) and (4) were added with culture medium; the area in the red dotted frame was treated with 5 min exposure to 808 nm laser. **b** Characterization of intracellular free radical and its oxidation products after exposure to 808 nm laser by DCFH-DA assay and **c** the corresponding quantitative fluorescence intensity. *p < 0.05, **p < 0.01 compared to the one treated with Bi_2_Se_3_@ZnPc NPs at the same condition. **d** Percentage of GSH amount in HepG2 cells treated with Bi_2_Se_3_@AIPH NPs (in normoxic and hypoxic atmosphere). *p < 0.05, **p < 0.01 compared to the one in the control cells. *n* = 4

Having validated the photothermal and free radical generation ability in aqueous solution, to further verify the dual-ability of Bi_2_Se_3_@AIPH at the cellular level, the behavior of Bi_2_Se_3_@AIPH in human hepatoma HepG2 cells was also examined. First, the cell uptake capability of Bi_2_Se_3_@AIPH was studied by loading Nile red (red fluorescence) in the NPs. The CLSM result in Fig. S9a, b visually shows that the red fluorescence from Nile red is noticeable after incubation for 1 h and becomes more distinct at 4 h. Meanwhile, the quantitative data in Fig. S9c by detecting the amount of Bi element in the HepG2 cells appear same discipline as the result above and the cell uptake amount of Bi_2_Se_3_@AIPH reaches 54.79 ng Bi/10^4^ cells at 1 h and up to 83.54 ng Bi/10^4^ cells at 4 h. Both the two ways verify the Bi_2_Se_3_@AIPH NPs can enter the HepG2 cells well. Then photothermal effect at the cellular level is also evaluated by HepG2 cells. As displayed in Fig. 2a, the temperature in the HepG2 cell culture medium co-incubated with Bi_2_Se_3_@AIPH exhibits significant increase, while the temperature in the control group shows almost no change, similarly to the ones without irradiation. This result verifies the photothermal conversion ability of Bi_2_Se_3_@AIPH in the HepG2 cells. The hyperthermia has been proved to cause direct cell death by many studies [4, 5, 39, 51].

Simultaneously, to evaluate the effect caused by the free radical in the HepG2 cells, dichlorofluorescein diacetate (DCFH-DA) assay was employed. According to the literature [43], free radical (R^·^) can convert to (RO^·^) under normoxic atmosphere. And intracellular free radicals (R·) or reactive oxygen species (RO·) could react with DCFH without fluorescence signal to produce DCF with green fluorescence [52]. To better illustrate the ability of producing toxic oxidation products of Bi_2_Se_3_@AIPH, we hired the traditional photosensitizer-zinc phthalocyanine (ZnPc) similarly loaded in the Bi_2_Se_3_ NPs as the contrast, which could produce ^1^O_2_ under the irradiation of 660 nm laser. When it works, ZnPc firstly releases under the exposure of 808 nm laser and then generates ^1^O_2_ under the irradiation of 660 nm laser. As shown in Fig. 2b, c, without irradiation, both of the cells treated with Bi_2_Se_3_@AIPH and Bi_2_Se_3_@ZnPc show indistinctive green fluorescence. While under the irradiation (Bi_2_Se_3_@AIPH group: 808 nm laser, 0.7 W cm^−2^, 5 min; Bi_2_Se_3_@ZnPc group: 808 nm laser, 0.7 W cm^−2^, 5 min and then 660 nm laser, 300 mW cm^−2^, 3 min), the conspicuous green fluorescence signals are observed in both of the groups at the normoxic atmosphere, but at the hypoxic atmosphere, only Bi_2_Se_3_@AIPH group displays similar fluorescence intensity as the one in the normoxic atmosphere, the contrast group shows almost no fluorescence signal. This result demonstrates that Bi_2_Se_3_@AIPH can produce free radicals or its oxidation products whether at the normoxic or hypoxic atmosphere under the irradiation, which is important for the hypoxic tumor therapy.

According to the literature [42, 43], free radicals or reactive oxygen species not only kill tumor cells directly but also induce oxidative stress. As one of the most important parameter in antioxidant system, the amount of glutathione (GSH) was tested by Ellman’s reagent to further reflect the effect of free radical. The result in Fig. 2d shows the intracellular GSH amount treated with Bi_2_Se_3_@AIPH greatly decreases compared to the control group under the exposure of 808 nm laser (GSH level: 15.6% in normoxic atmosphere and 16.3% in hypoxic environment). The pronounced GSH depletion of Bi_2_Se_3_@AIPH demonstrates the destruction of the redox balance and the increasing oxidative stress. When oxidative stress is too much to self-regulate recovery, it may also cause disorders and damages [53]. Dual effect of hyperthermia and free radicals could be a powerful weapon to induce tumor cell death whether at the normoxic or hypoxic atmosphere.

**Fig. 3:**
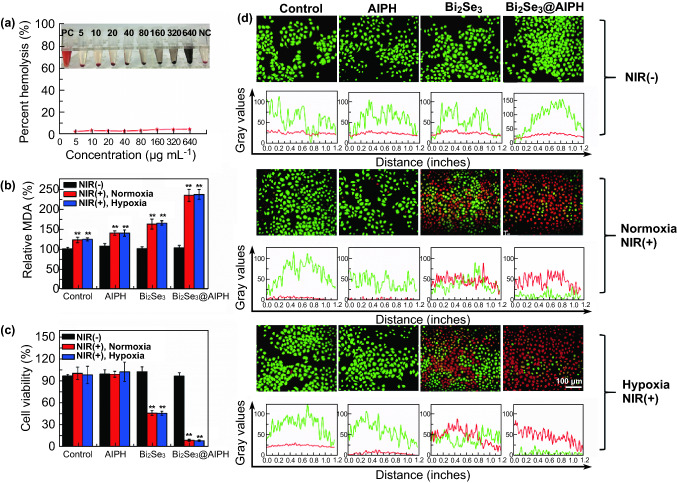
Evaluation of biocompatibility and cytotoxicity. **a** Hemolytic percentages of red blood cells incubated with Bi_2_Se_3_@AIPH NPs at the concentrations of 5, 10, 20, 40, 80, 160, 320, and 640 µg mL^−1^ for 2 h. Inset: hemolysis photograph after centrifugation. **b** Relative MDA percentage after treatment with PBS, AIPH, Bi_2_Se_3_, and Bi_2_Se_3_@AIPH. **c** Cytotoxicity to HepG2 cells treated with PBS, AIPH, Bi_2_Se_3_, and Bi_2_Se_3_@AIPH in normoxic and hypoxic atmosphere. **d** Apoptosis images and gray value analysis of HepG2 cells treated with PBS, AIPH, Bi_2_Se_3_, and Bi_2_Se_3_@AIPH in normoxic and hypoxic atmosphere. The irradiation to HepG2 cells was all conducted by the 808 nm laser (1 W cm^−2^, 5 min). *p < 0.05, **p < 0.01 compared to the one in the control cells. *n* = 4

Encouraged by the excellent effect of hyperthermia and free radical in vitro, we studied the in vitro therapeutic effect of Bi_2_Se_3_@AIPH NPs. Before that, the biocompatibility of the material was tested by the hemolytic and MTT experiments. First, the result of hemolytic experiment in Fig. 3a shows that no obvious hemolysis effect appears and hemolysis ratio of NPs at all concentrations (0–640 µg mL^−1^) is less than 5%, indicating good blood compatibility. Moreover, in Figs. S10 and S11, the HepG2 cell viability is all over 90% after incubated with LA (0–100 µg mL^−1^), AIPH (0–60 µg mL^−1^), and Bi_2_Se_3_@AIPH (0–400 µg mL^−1^), indicating no obvious toxicity of LA, AIPH, and Bi_2_Se_3_@AIPH to HepG2 cells at the working concentration. All of these results demonstrate excellent biocompatibility of our material.

Moreover, the most important ability of Bi_2_Se_3_@AIPH NPs, cytotoxicity, is explored. Since oxygen is severely deficient in most tumor, we studied the toxicity in both normoxic and hypoxic atmosphere. First, the cell membrane damage was evaluated by measuring the methane dicarboxylic aldehyde (MDA, one of end products of lipid peroxidation), since hyperthermia and free radical (mainly) would destroy the cell membrane to destroy cells. The results in Fig. 3b show that whether in normoxic and hypoxic atmosphere, the MDA contents of Bi_2_Se_3_@AIPH are nearly 1.9-fold of the control group and 1.4-fold of Bi_2_Se_3_ under the irradiation, which verifies the destruction ability of Bi_2_Se_3_@AIPH NPs. Meanwhile, as shown in Fig. 3c, the cytotoxicity was conducted and the results show that whether in normoxic or hypoxic condition, both of cell viabilities in the group treated with Bi_2_Se_3_ under the irradiation are about 45%, which proved the killing effect of hyperthermia. And the living cells in the group treated with Bi_2_Se_3_@AIPH greatly reduced under the irradiation of 808 nm laser (cell viability: 8.5% in normoxic condition and 8.0% in hypoxic condition), which also verifies the outstanding synergistic killing effect of hyperthermia and toxic free radical. Meanwhile, the acridine orange/ethidium bromide (AO/EB) dual staining was employed to visually study the apoptosis of HepG2 cells. Since AO dye could stain both live and dead cells, while EB dye just stains the cells without intact membrane, live cells are marked as green, whereas apoptotic cells present red or jacinth. As seen in Fig. 3d, without irradiation, basically all the cells are alive. While with irradiation, the groups appear similar results whether at normoxic or hypoxic atmosphere. Apoptosis in the groups treated with PBS and AIPH is basically invisible, while the one in the Bi_2_Se_3_ group is pronounced, which verified the effect of hyperthermia generated from the NIR light absorption of Bi_2_Se_3_. Importantly, red and jacinth fluorescence in most HepG2 cells presents in the group treated with Bi_2_Se_3_@AIPH NPs and the green fluorescence is rarely seen, which demonstrates effects of dual effect. The gray value of each apoptosis image is also presented below each image.

### In Vitro and In Vivo CT Imaging

**Fig. 4:**
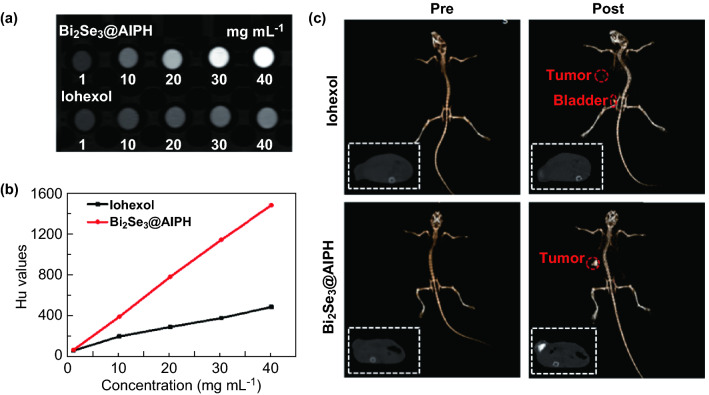
In vitro and in vivo CT imaging. **a** The in vitro CT imaging pictures and **b** quantitative HU values of Bi_2_Se_3_@AIPH NPs and iohexol at the concentrations of 1, 10, 20, 30, and 40 mg mL^−1^. **c** The 2D sectional imaging and 3D reconstruction pictures before and after the injection of Bi_2_Se_3_@AIPH NPs and iohexol

Encouraged by the amazing killing effect in the HepG2 cells, the in vivo behavior is also studied. As we know, imaging guide approach is essential for light-triggered therapy. Owing to the high X-ray attenuation coefficient of Bi [Bi (5.74) > Au (5.16) > Pt (4.99) > Ta (4.3) > I (1.94 cm^2^ kg^−1^) at 100 keV) [47], Bi_2_Se_3_@AIPH has the potential of CT imaging with high quality. First, we study the in vitro CT imaging of Bi_2_Se_3_@AIPH NPs and use clinical CT contrast agent (iohexol) as the control group. And the result in Fig. 4a, b showed that intuitive CT image of Bi_2_Se_3_@AIPH NPs at the same concentration is much brighter than iohexol. The HU values characterizing CT signal intensity present a linear increase with the concentrations of Bi_2_Se_3_@AIPH (average X-ray attenuation coefficient is 37.77 HU mL mg^−1^), which is much higher than commercial contrast agents iohexol (Average X-ray attenuation coefficient is 14.25 HU mL mg^−1^).

Based on the great in vitro behavior of Bi_2_Se_3_@AIPH, in vivo CT image of Bi_2_Se_3_@AIPH is investigated using an X-ray CT imaging system. First, we tested the in vivo CT imaging capability of Bi_2_Se_3_@AIPH compared to the iohexol. Both two-dimensional (2D) sectional imaging and three-dimensional (3D) reconstruction are shown in Fig. 4c. For both the groups (intratumorally injected with Bi_2_Se_3_@AIPH and iohexol), the CT signal intensity in the tumor region apparently increases compared to the ones before the injection. Moreover, the contrasts of tumors in the mice injected with Bi_2_Se_3_@AIPH are much stronger than the one injected with iohexol. And in the iohexol group, an obvious CT signal is found in the bladder, which indicated the rapid drainage of iohexol through the urine (20 min after the injection). We speculate that iohexol as a small molecule is easily washed out in vivo, which may lower the quality of CT imaging. Simultaneously, we also studied the CT imaging effects before and after (3, 6, and 24 h) intravenous injection of Bi_2_Se_3_@AIPH NPs. The representative 3D reconstruction, 2D sectional imaging pictures, and the quantitative average CT values at 0, 3, 6, and 24 h are shown in Fig. S12a, b. The results show that the CT brightness at 3, 6, and 24 h at the tumors is enhanced by 90%, 170%, and 60% comparing to the ones at 0 h, which demonstrates that Bi_2_Se_3_@AIPH NPs could be a good imaging enhancer and may avoid the rapid drainage problem of commercial contrast agents. Moreover, the CT value peaks at 6 h post-injection and has the highest contrast. Simultaneously, the biodistributions of Bi element at these time point were also measured by ICP-AES, and the Bi contents in the tumors are 10.69, 18.07, and 6.6 μg g^−1^ tumor tissue at 3, 6, and 24 h, respectively. This result is basically consistent with the CT values of tumors at different time points mentioned above. Moreover, the biodistribution result also shows that the Bi contents are mainly accumulated in the liver and spleen (the major reticuloendothelial system organ in the body), which is consistent with the results reported in the literature [47]. We believe the outstanding performance of Bi_2_Se_3_@AIPH NPs in CT imaging could give guidance to tumor therapy.

### In Vivo Antitumor Efficacy

**Fig. 5:**
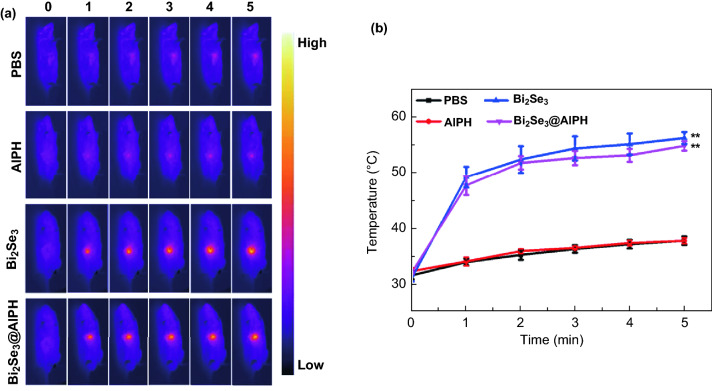
**a** In vivo photothermal images and **b** the corresponding temperature change in mice after the injection of PBS, AIPH, Bi_2_Se_3_, and Bi_2_Se_3_@AIPH under 808 nm laser irradiation (1 W cm^−2^, 5 min). *p < 0.05, **p < 0.01

Except the high CT imaging, thermal image is also an assist for judging the tumor region. The result in Fig. 5a, b shows that the mice in the groups injected with PBS and AIPH have the similar thermal image effects. And the temperatures of the tumor regions in both groups only slightly increase, and the highest temperature is just about 37 °C. While for the mice in the groups injected with Bi_2_Se_3_@AIPH and Bi_2_Se_3_, the temperatures in the tumor region also have the similar thermal discipline and both are over than 50 °C at 5 min, which is enough for the hyperthermia therapy and melting LA in the Bi_2_Se_3_@AIPH to produce free radical by thermal decomposition.

**Fig. 6:**
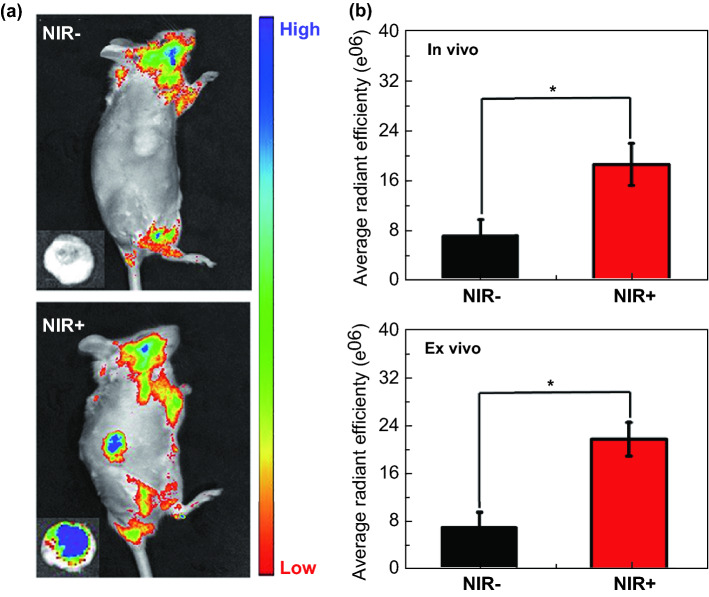
**a** Fluorescence imaging photographs of the mice with and without 808 nm irradiation. Inset: the corresponding tumor. **b** Quantitative average radiant efficiency of the tumors in and ex vivo. Both mice were injected with Bi_2_Se_3_@AIPH NPs. *p < 0.05, **p < 0.01

The thermal effect was verified by the temperature increase in the mice. Meanwhile, the generated free radical was also studied by in vivo fluorescence imaging. Dihydrorhodamine is easily reduced to rhodamine by the oxidation of free radical [54, 55]. Dihydrorhodamine has no fluorescence signal, while rhodamine could be detected by in vivo fluorescence imaging system. Thus, dihydrorhodamine was used to explore the free radical effect and the result is displayed in Fig. 6a–b. The mice were both injected with the material. The one irradiated by 808 nm laser shows obvious fluorescence signal in the tumor site, while the fluorescence signal in the one without irradiation is hardly seen. And the ex vivo pictures also verify this result, and the fluorescence signal is a little higher than the result in vivo. The in vivo and ex vivo quantitative data are consistent with the above results.

**Fig. 7:**
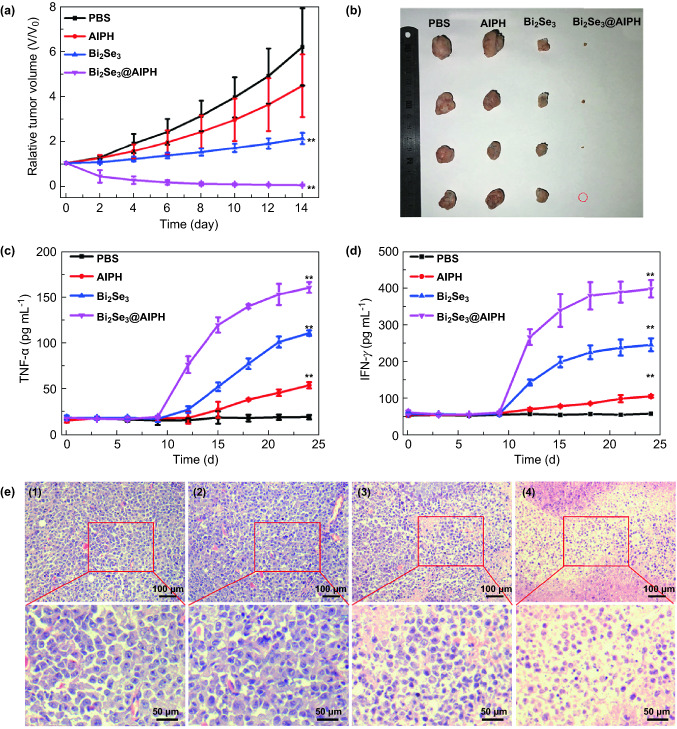
**a** Relative tumor volume changes in 14 days. **b** Photographs of the excised tumors after 14 days of treatment. **c** The TNF-α and **d** IFN-γ level during the treatment measured every 3 days. The vaccination of H22 tumor cells marked as 0 day, and the treatment starts at day 9. **e** Images of tumor sections by H&E staining. *p < 0.05, **p < 0.01

Most importantly, the tumor suppressive ability of Bi_2_Se_3_@AIPH NPs is studied. After being irradiated by 808 nm laser (1 W cm^−2^, 5 min), we measured the body weight and tumor size every 2 days. As shown in Fig. S13, the body weight change curve in the group injected with Bi_2_Se_3_@AIPH is similar to the other groups (PBS, AIPH, and Bi_2_Se_3_), which indicates that the Bi_2_Se_3_@AIPH has no obvious damage to the mice. Furthermore, the relative tumor volume change in 14 days in Fig. 7a indicates that the Bi_2_Se_3_@AIPH NPs has great killing effect for the tumor than other groups. At Day 14, the mice were sacrificed and tumors taken out from the mice were pictured and measured. As shown in Fig. 7b, the tumor in the Bi_2_Se_3_@AIPH group is really small and even disappears, which indicates the significant therapeutic effect of Bi_2_Se_3_@AIPH NPs. And the tumor inhibition rate in the Bi_2_Se_3_@AIPH group is as high as 99.6%, while tumor inhibition rate in the Bi_2_Se_3_ group is only 65.8% under the same condition, which demonstrates the better killing effect of hyperthermia and free radical.

Moreover, according to the result in Fig. 7a, the tumor did not completely diminish after the irradiation of 808 nm laser; however, the tumor volume decreased continuously in the next several days without further irradiation. We speculated that the immune response would be responsible for this phenomenon. In addition to the known dual killing of PTT and oxygen-independent PDT, whether the Bi_2_Se_3_@AIPH NPs trigger the immune response to enhance the treatment effect is also studied. As the representative markers for immune response, the cytokine TNF-α and IFN-γ in serum were detected by corresponding Mouse ELISA Kit. The result in Fig. 7c, d showed that both the TNF-α and IFN-γ levels in the Bi_2_Se_3_ and Bi_2_Se_3_@AIPH group significantly increase compared to the PBS group after the treatment. These up-regulations demonstrate both of Bi_2_Se_3_ and Bi_2_Se_3_@AIPH treatments could induce the immune response in mice. And initiating immune response could help recognize, track down, and destroy any remaining tumor cells [56–59], which accounts for the further reduction in tumor volume after one exposure to NIR laser. Moreover, the secretion amount of TNF-α and IFN-γ in the Bi_2_Se_3_@AIPH group is significantly higher than the ones in the Bi_2_Se_3_ group (1.45-fold for TNF-α and 1.63-fold for IFN-γ), which demonstrates the introduction of AIPH into the Bi_2_Se_3_@AIPH NPs could effectively enhance immune response in the following PDT treatment and further achieve better treatment effect.

Furthermore, the visual characterization of tumor damage is also achieved by H&E staining. As displayed in Fig. 7e, pathological abnormalities are hardly found in the PBS and AIPH group, but the obvious characteristics of necrosis and apoptosis (cell shrinkage and karyolysis) have appeared in the Bi_2_Se_3_ and Bi_2_Se_3_@AIPH group, and the cell activity in the Bi_2_Se_3_@AIPH group greatly reduced, which illustrates the synergistic therapeutic effect. And the toxicities to main organs (heart, liver, spleen, lung, and kidney) were also studied by H&E staining, and the results of all the groups in Fig. S14 show no pathological abnormalities, which demonstrates the good biocompatibility of Bi_2_Se_3_@AIPH.

## Conclusions

In summary, we designed a facile material (Bi_2_Se_3_@AIPH NPs) which can simultaneously produce hyperthermia and oxygen-independent free radical by a single 808-nm laser irradiation. The loading amount of AIPH in Bi_2_Se_3_ NPs is 10.2%. The results show that the photothermal conversion efficiency of Bi_2_Se_3_@AIPH is 31.2% and free radical generated from thermal decomposition is captured by POBN and DMPO. And in vitro and in vivo photothermal effect and free radical generation of Bi_2_Se_3_@AIPH have been proved by fluorescence imaging and thermal imaging, respectively. Then, by MTT and AO/EB assays, excellent killing efficiency of dual functions has been verified (both over 90% killing rates at the normoxic and hypoxic atmosphere). Furthermore, in vivo CT imaging capability of Bi_2_Se_3_@AIPH NPs (37.77 HU mL mg^−1^) is much better than clinically used iohexol (14.25 HU mL mg^−1^), which is important for imaging-guided tumor therapy. Most importantly, the tumor growth inhibitory rate is 99.6% compared to PTT alone (65.8%), demonstrating outstanding overall therapeutic efficacy of Bi_2_Se_3_@AIPH NPs. We believe this facile multifunctional material could provide some references for phototherapy.

## Electronic supplementary material

Below is the link to the electronic supplementary material.

Supplementary material 1 (PDF 942 kb)
