# Supplementary material for: Single NIR Laser-Activated Multifunctional Nanoparticles for Cascaded Photothermal and Oxygen-Independent Photodynamic Therapy
Source: Nanomicro Lett. 2019 Aug 19;11:68. doi: 10.1007/s40820-019-0298-5 (PMC7770756; doi:10.1007/s40820-019-0298-5)
Supplement: Supplementary file 1 — Supplementary material 1 (PDF 942 kb) [file 40820_2019_298_MOESM1_ESM.pdf]

Supporting Information for

Single NIR Laser-Activated Multifunctional Nanoparticles for Cascaded Photothermal and Oxygen-Independent Photodynamic Therapy

Xiaomin Li[1], Yang Liu[1], Fei Fu[2], Mingbo Cheng[1], Yutong Liu[1], Licheng Yu[1], Wei Wang[1], Yeda Wan[2], Zhi Yuan[1, 3, *]

[1]Key Laboratory of Functional Polymer Materials of Ministry of Education, College of Chemistry, Nankai University, Tianjin 300071, People's Republic of China

[2]Department of Radiology, Tianjin Hospital, Tianjin 300210, People's Republic of China

[3]Collaborative Innovation Center of Chemical Science and Engineering, Nankai University, Tianjin 300071, People's Republic of China

*Corresponding author. E-mail: zhiy@nankai.edu.cn (Zhi Yuan)

S1 Synthesis of Bi_2Se_3 NPs

Synthesis of Bi_2O_3: The Bi_2O_3 was synthesized according to previous methods [S1–S3]. Typically, 0.364 g $Bi(NO_3)_3·5H_2O$ was firstly dissolved by HNO_3 solution (10 mL, 1 M), followed by the adding of 0.108 g NaOH, 0.6 g PVP and 50 mL EG solution. After thorough dissolution under stirring, the mixture was transferred to the stainless steel autoclave, and reacted at 150 °C for 3 h. After cooling down to room temperature, the reaction solution was centrifuged and washed by DI water for 4 times. The final milk-white product was dried by lyophilization and placed in a dryer.

Synthesis of Bi_2Se_3: The Bi_2Se_3 was prepared by previous methods with small modification [S2]. Briefly, 0.2 g Na_2SeO_3 and 0.6 g ascorbic acid were dissolved in 30 mL DI water, followed by the adding of the above Bi_2O_3 NPs dissolved in 10 mL DI water. Similarly, the mixture reacted in the autoclave (150 °C, 12 h). Then the reaction solution was purified by centrifugation and dialysis. The final black product was dried using the same method as Bi_2O_3.

S2 Cell Uptake

To measure the cell uptake of Bi_2Se_3@AIPH, the Nile red-labeled Bi_2Se_3@ AIPH was prepared using similar method as preparation of Bi_2Se_3@AIPH. Briefly, 0.2 g AIPH,

0.15 g LA and 0.02 g Nile red was dissolved in mixed solvent (DI water/methanol=1:1). And then 3 mg Bi_2Se_3 was added into the mixture and continued to react for 3 days. And the post processing is completely the same as the one in Synthesis of Bi_2Se_3@AIPH. Then the cells were seeded into Petri-dish and incubated for 24 h. And then the Nile red-labeled Bi_2Se_3@ AIPH (40 μg mL^{-1}) was added and incubated for 1 and 4 h, respectively. After that, the cells were washed by PBS for three times, followed by staining with Lysotracker Green. Then after the HepG2 cells were fixed by 4% paraformaldehyde, the cells were stained with DAPI which would dye the cell nuclei with blue fluorescence. And then Laser Scanning Confocal Microscopy (CLSM) is employed to visually observe the uptake of Bi_2Se_3@AIPH. Meanwhile, to obtain quantitative result, after the incubation and washing, the cells were dissolved by aqua regia and dilute 5 times. After centrifuging, the Bi element of the supernatant was detected by Inductively Coupled Plasma-Atomic Emission Spectrometry (ICP-AES).

S3 Hemolytic and Stability Test

The fresh blood (1 mL) collected from healthy ICR mice using anticoagulant tube were diluted by 5 mL PBS solution. Through centrifugation (1200 r, 5 min, 4 times), the red blood cells (RBCs) were separated and rinsed, and finally dispersed in 10 mL PBS solution. 100 μL Bi_2Se_3@AIPH solutions (5, 10, 20, 40, 80, 160, 320, and 640 μg mL^{-1}) were added into 200 μL diluted RBCs solution, respectively. And after 6 h incubation at 37 °C and centrifugation once again, the supernatants were detected by UV-Vis spectrum. And the absorbance intensity at 540 nm was used to estimate the level of hemoglobin. The negative control and positive control were achieved by mixed PBS and 2% Triton-100 with diluted RBCs solution. The percent hemolysis of RBCs was calculated according to the literature [S4].

S4 Cytotoxicity of LA and AIPH

The cytotoxicity of LA at different concentrations (0, 3, 10, 20, 30, 40, 50, 60, 70, 80, 90, and 100 μg mL^{-1}) and AIPH at different concentrations (0, 4, 10, 20, 30, 40, 50, 60, 70, 80, 90, and 100 μg mL^{-1}) was tested by MTT assay. The working concentrations used in the cytotoxicity experiment were 3 and 4 μg mL^{-1}.

S5 Supplementary Figures

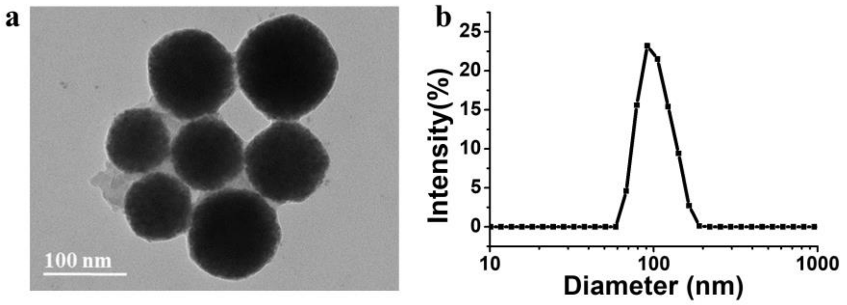

Fig. S1 a TEM image and **b** the hydrodynamic diameter of Bi_2O_3 NPs

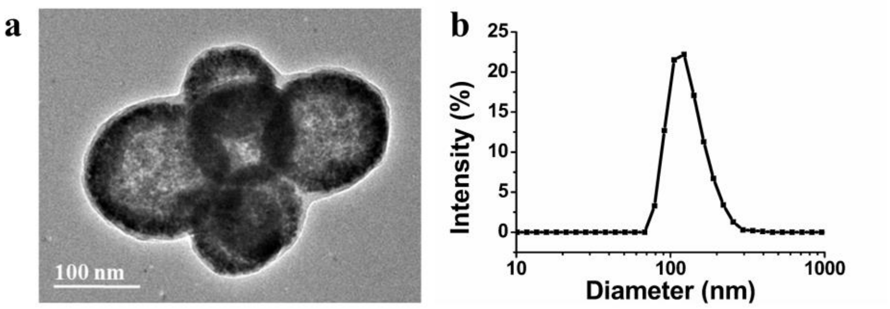

Fig. S2 a TEM image and **b** hydrodynamic diameter of Bi_2Se_3 NPs

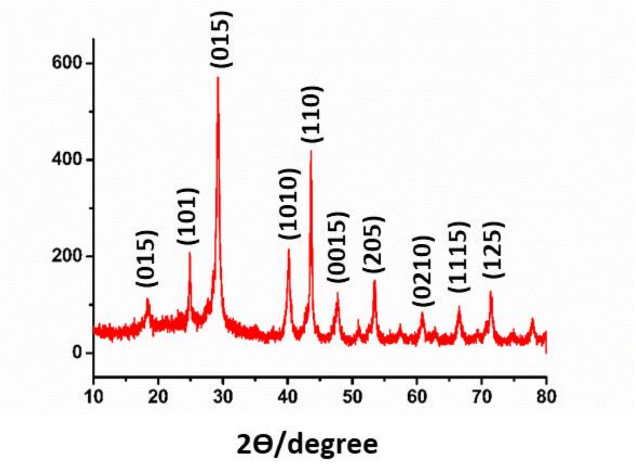

Fig. S3 XRD pattern of Bi_2Se_3@AIPH NPs

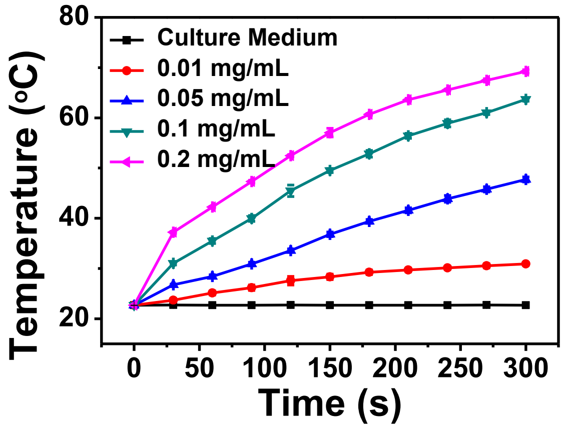

Fig. S4 Temperature change evaluation of Bi_2Se_3 NPs at the concentrations of 0, 0.01, 0.05, 0.1 and 0.2 mg mL^{-1} under the exposure to 808 nm laser (1 W cm^{-2}, 5 min)

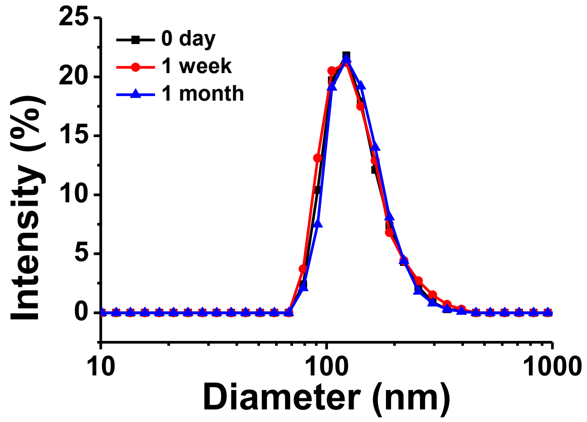

Fig. S5 Hydrodynamic diameter of Bi_2Se_3@AIPH at 0 day, 1 week and one month

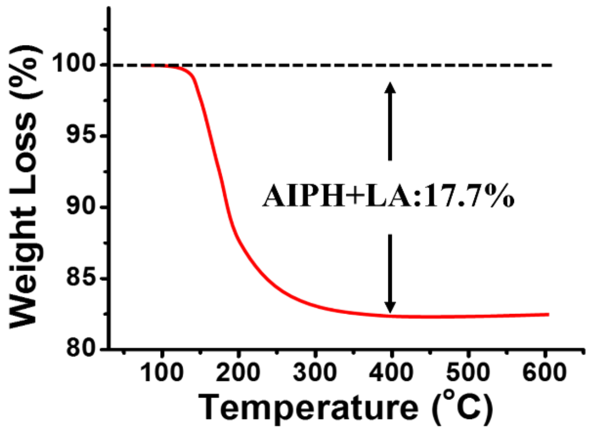

Fig. S6 TGA data of Bi_2Se_3@AIPH

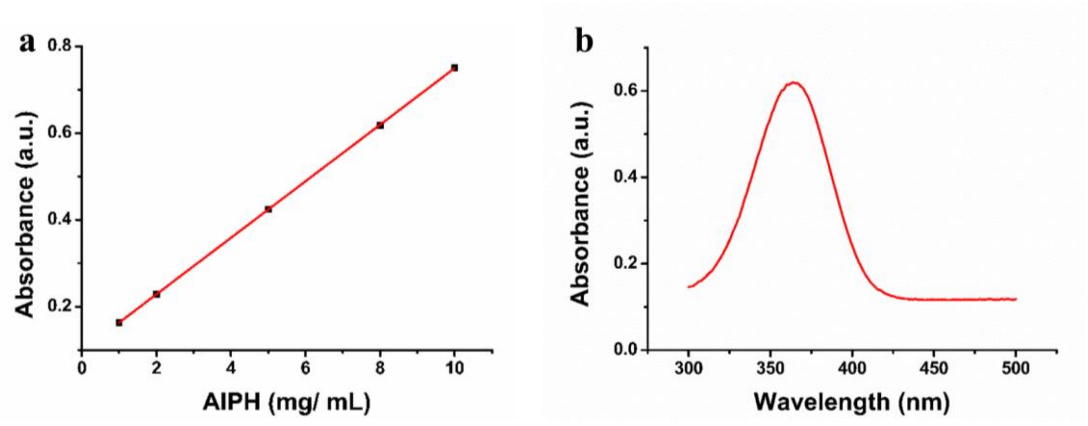

Fig. S7 a Standard curve of AIPH (concentration range: 1-10 mg mL^{-1}). **b** UV-Vis spectrum of AIPH not loaded in Bi_2Se_3@AIPH

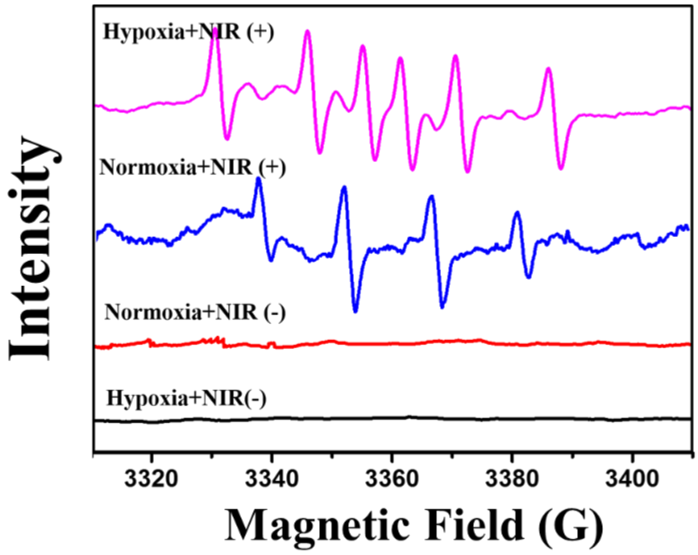

Fig. S8 ESR spectrum of 50 mM DMPO in 0.1 mg mL^{-1} Bi_2Se_3@AIPH with or without irradiation at normoxic and hypoxic atmosphere

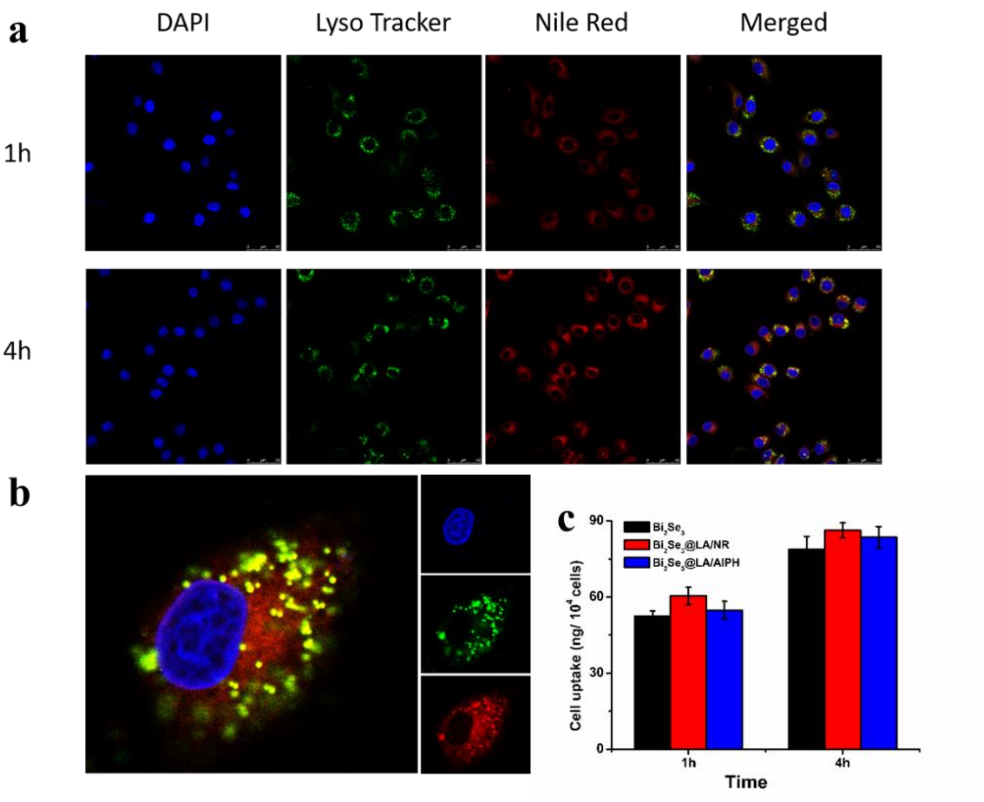

Fig. S9 The cell uptake of Nile red-labeled Bi_2Se_3@AIPH in 1 h and 4 h

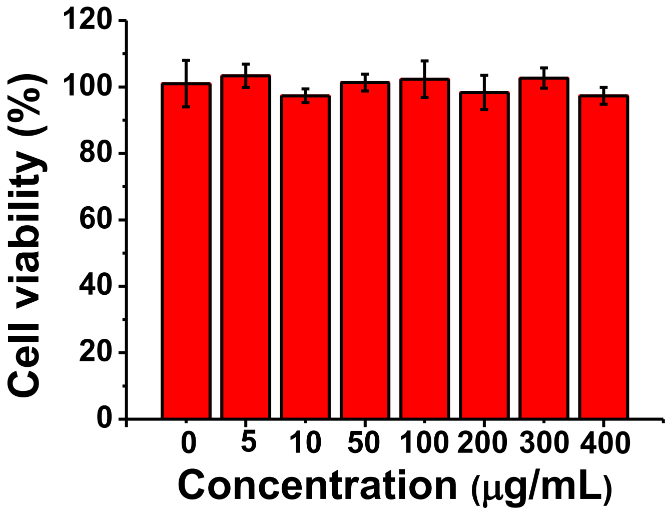

Fig. S10 The cytotoxicity of HepG2 cells treated with Bi_2Se_3 at the concentrations of 0-400 μg mL^{-1}

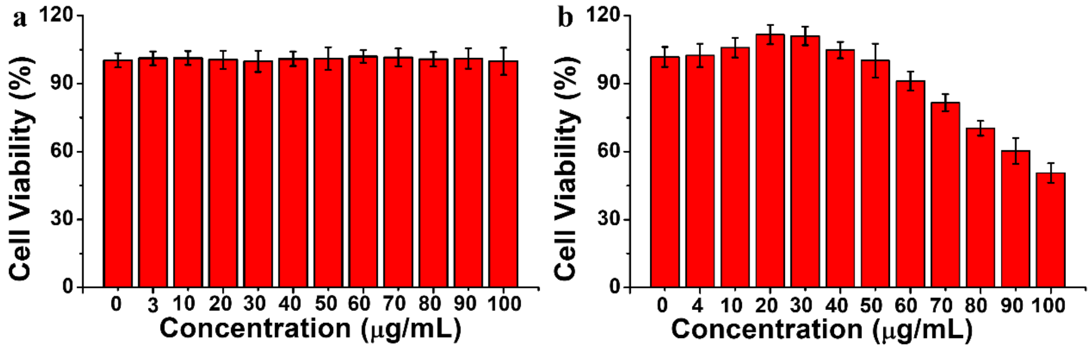

Fig. S11 The cytotoxicity of HepG2 cells treated with **a** LA and **b** AIPH at the concentrations of 0-100 μg mL^{-1}

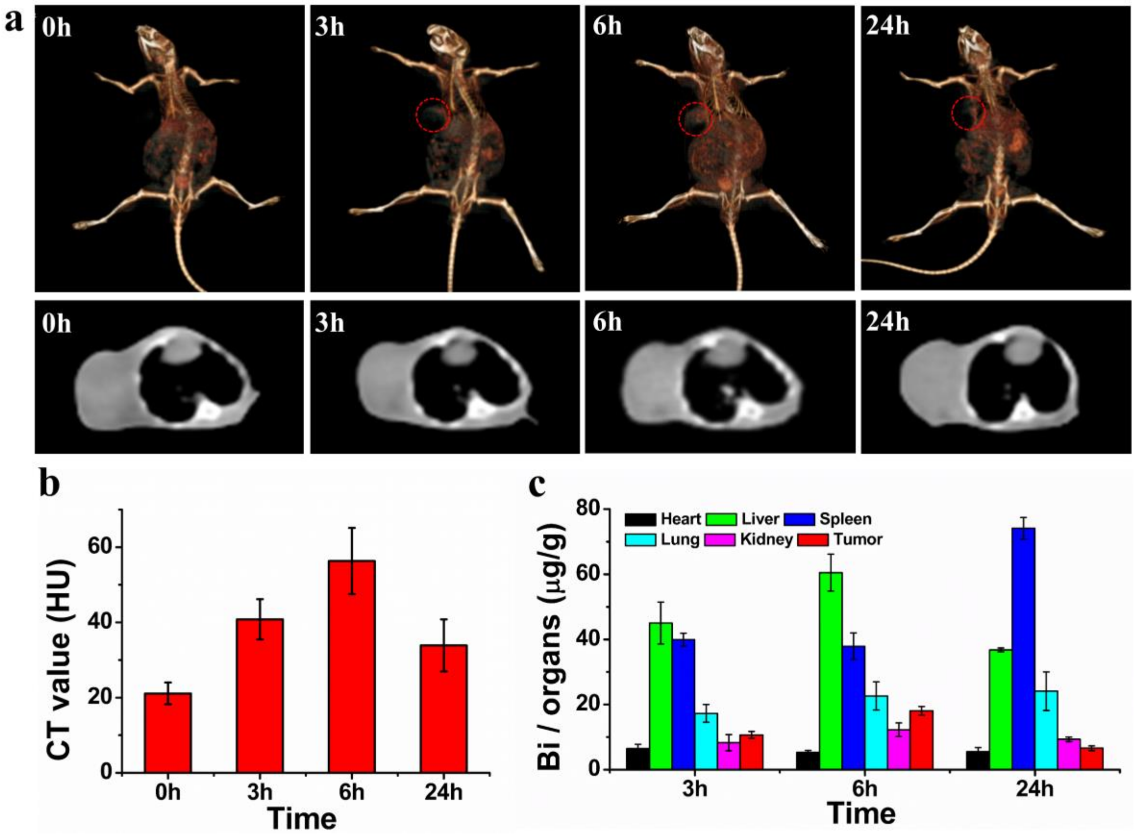

Fig. S12 CT imaging and biodistributions before (pre-injection) and after (3 h, 6 h, and 24 h) intravenous injection of Bi_2Se_3@AIPH. **a** representative 3D reconstruction and 2D imaging pictures, and **b** average CT values at 0 h, 3 h, 6 h, and 24 h. **c** The biodistributions of Bi element in heart, liver, spleen, lung, kidney and tumor at 3 h, 6 h, and 24 h. The red circles indicate tumor regions n=3

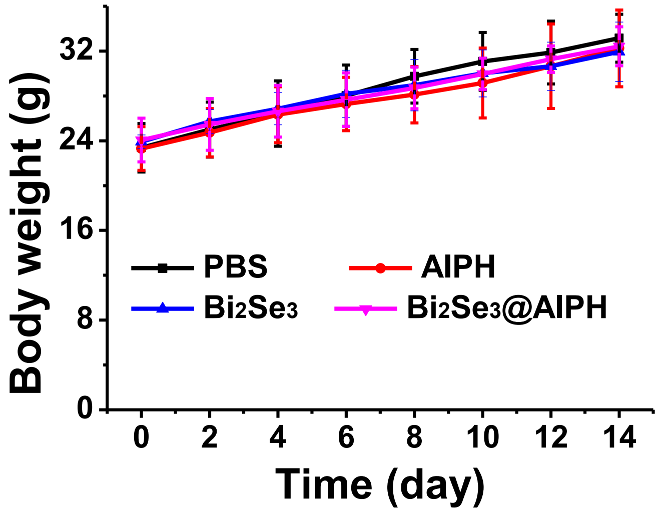

Fig. S13 Body weight change of mice in 14 days injected with PBS, AIPH, Bi_2Se_3 and Bi_2Se_3@AIPH measured every two days. Data above are presented as means with standard deviations (n = 4) (mean ± SD)

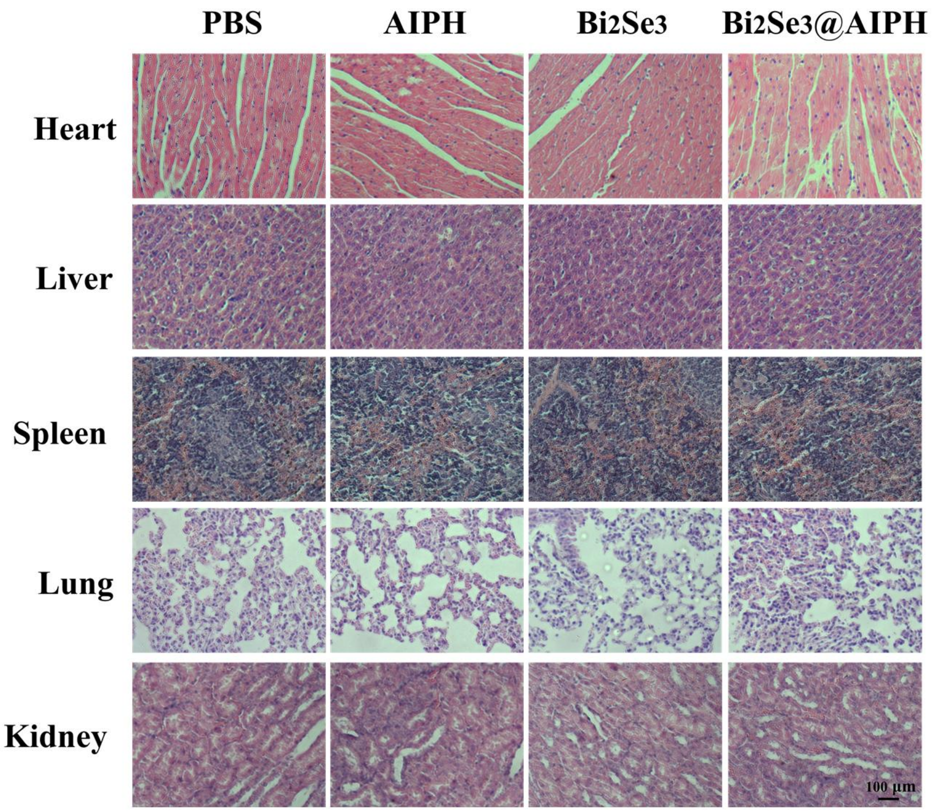

Fig. S14 Representative H&E pictures of major organs (heart, liver, spleen, lung, and kidney) after the 14-day treatment (injected with PBS, APIH, Bi_2Se_3, Bi_2Se_3@AIPH via tail vein and irradiated by 808nm laser)

Suppplementary References

[S1] F. Qin, H. Zhao, G. Li, H. Yang, J. Li et al., Size-tunable fabrication of multifunctional Bi_2O_3 porous nanospheres for photocatalysis, bacteria inactivation and template-synthesis. Nanoscale **6**(10), 5402 (2014). http://doi.org/10.1039/c3nr06870f

[S2] Z. Li, J. Liu, Y. Hu, K.A. Howard, Z. Li et al., Multimodal imaging-guided antitumor photothermal therapy and drug delivery using bismuth selenide spherical sponge. ACS Nano **10**(10), 9646 (2016). http://doi.org/10.1021/acsnano.6b05427

[S3] Z. Li, Y. Hu, M. Chang, K.A. Howard, X. Fan, Y. Sun, F. Besenbacher, M. Yu, Highly porous PEGylated Bi 2 S 3 nano-urchins as a versatile platform for in vivo triple-modal imaging, photothermal therapy and drug delivery. Nanoscale **8**(35), 16005 (2016). http://doi.org/10.1039/C6NR03398A

[S4] Y. Shao, C. Shi, G. Xu, D. Guo, J. Luo, Photo and Redox Dual Responsive Reversibly Cross-Linked Nanocarrier for Efficient Tumor-Targeted Drug Delivery. ACS Appl. Mater. Interfaces **6**(13), 10381 (2014). http://doi.org/10.1021/am501913m
